# Bioresorbable hyaluronic acid alginate hydrogel use in total ankle arthroplasty to control post-operative scarring

**DOI:** 10.1093/jscr/rjaf533

**Published:** 2025-07-14

**Authors:** Jay S Badell

**Affiliations:** Fellowship Trained Foot and Ankle Surgeon, Hancock Orthopedics, Hancock Health, 801 N. State St. Suite 2100, Greenfield, IN 46140, United States

**Keywords:** total ankle replacement, ankle arthroplasty, hyaluronic acid, alginate, VersaWrap, VersaWrap tendon protector

## Abstract

End-stage ankle arthritis can be a debilitating condition, with ankle replacement as an avenue for significant pain relief and functional return to activity. However, peri-prosthetic scarring and ectopic bone formation are commonly observed following joint replacement surgery, and several methods of treatment exist to address these conditions post-operatively, with varied success. Maintenance of ankle joint range of motion has been shown to lead to lower pain and higher patient satisfaction following ankle replacement. Presented in this study is the use of a hyaluronic acid alginate hydrogel at the time of index surgery to decrease the risk of peri-prosthetic scarring.

## Introduction

Total ankle replacement (TAR) is quickly gaining popularity as a surgical option for end-stage ankle arthritis. Technology over recent years has improved, with greater survivorship being reported in the literature [1]. However, a common post-operative problem with TAR, not unlike total knee, hip, or shoulder arthroplasty, is the formation of scar tissue and heterotopic bone over time [2–5]. These unwanted issues can be a painful condition and can significantly reduce range of motion and patient satisfaction [6]. The purpose of this study is to present the technique for use of a novel hydrogel sheet composed of hyaluronic acid (HA) and alginate in combination with total ankle replacement to mitigate post-operative scarring.

## Materials and methods

Any patient with end-stage ankle arthritis is a candidate for TAR. Several factors must be considered, such as age, BMI, comorbidities, functional status, and bone quality, among others [7]. Contraindications to ankle arthroplasty would include presence of a Charcot joint, poor bone stock, uncontrolled diabetes, and peripheral vascular disease that would put the patient at risk of wound healing complications. Indications for the use of a HA alginate hydrogel, VersaWrap (VersaWrap Tendon Protector; Alafair Biosciences, Austin, TX) include application on tendon and surrounding tissues, such a ligament or skeletal muscle; the device may also encounter implanted structures such as those used to conduct the joint replacement. VersaWrap can be applied during TAR procedures to address patients with peri-joint scarring or gutter impingement. Contraindications to the use of the hydrogel would include allergy to either HA, alginate, or aqueous citrate.

All patients planning to undergo TAR undergo initial conservative therapy, which could include one or several of the following, but not limited to: intraarticular steroid injection, platelet-rich plasma injection, bracing, and physical therapy. Once conservative measures are exhausted, pre-operative MRI and/or CT is routinely performed to evaluate the peri-articular bone quality as well as the soft tissue structures about the ankle. Patients who are acceptable candidates based on demographics, comorbidities, and imaging are provided the option of ankle replacement versus ankle fusion. If electing to proceed with ankle replacement, the use of patient-specific instrumentation may or may not be utilized as well.

## Technique

The example case presented is of a 69-year-old female who sustained a trimalleolar fracture with deltoid injury in 2017 and subsequently underwent open reduction internal fixation (ORIF). Progressive pain from post-traumatic arthritis limited her activity and ankle range of motion. She completed extensive conservative therapy for multiple years with bracing, injections, and physical therapy without relief. Ultimately, the patient elected to undergo TAR for definitive management.

Standard anterior approach is utilized to access the ankle joint. The ankle implant system is selected per surgeon preference. Very commonly, the surgeon will encounter scarring in the posterior ankle capsule following the removal of the tibial and talar bone resections. The author routinely performs a careful release of the posterior portion of the ankle capsule to allow greater range of motion about the ankle joint [8]. If significant soft tissue or bony impingement is noted in the ankle gutters, as was the case for this patient, debridement can also be performed to allow smooth range of motion.

**Figure 1:**
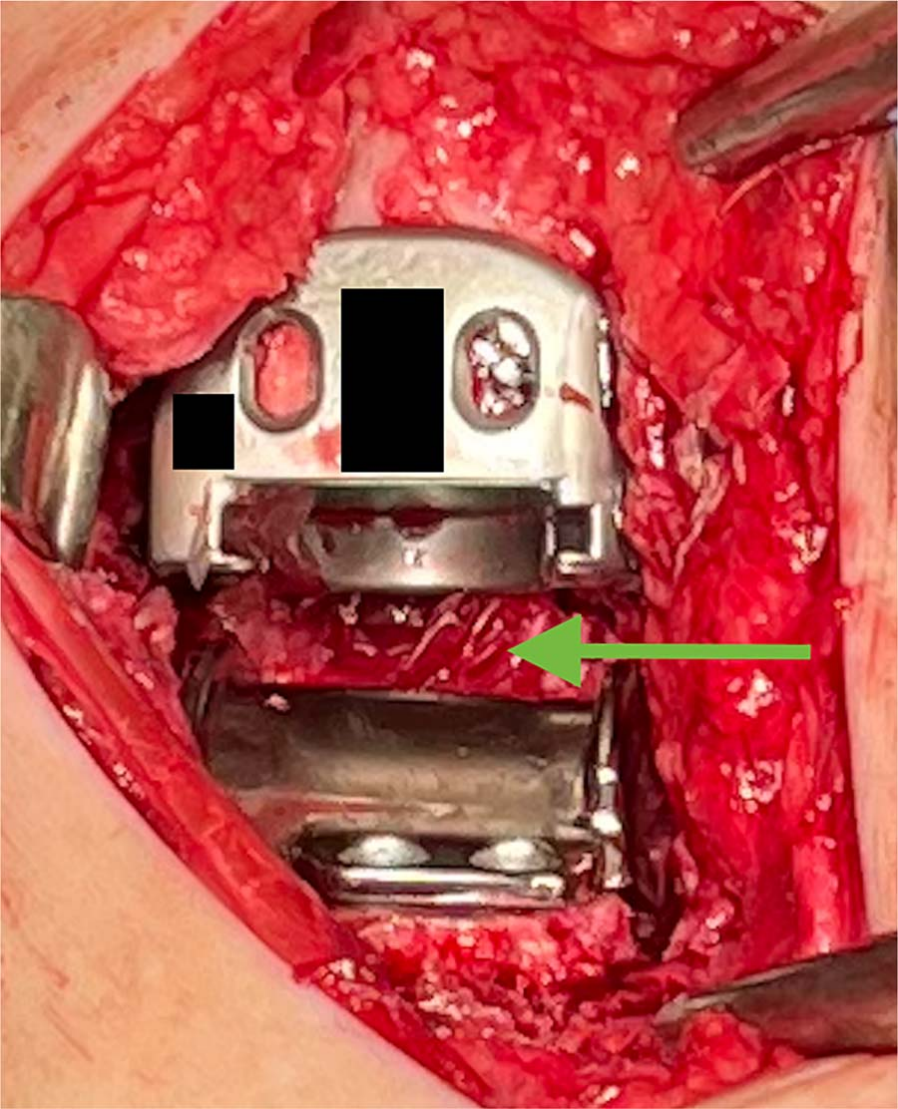
VersaWrap placed in the posterior ankle following capsular release.

**Figure 2:**
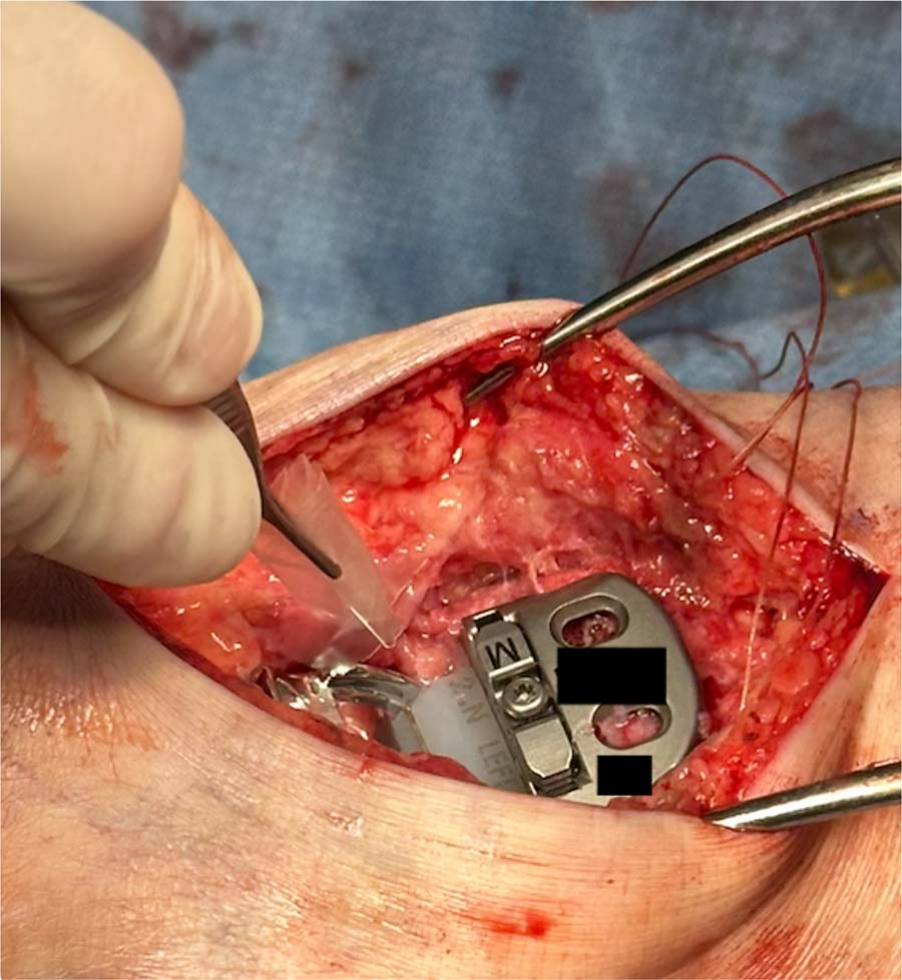
VersaWrap placed into medial ankle gutter.

The hydrogel sheet is composed of a biocompatible, bioresorbable HA alginate. The sheet is placed within the posterior ankle capsule after tibial and talar components have been implanted, but before final polyethylene insertion (Fig. 1). The aqueous citrate wetting solution is then added to the hydrogel sheet surface to activate. The goal of this application is to prevent return of scar tissue formation in the posterior ankle after the previous capsule release performed earlier in the surgery. Once all implants and poly have been finalized and just before closure, additional VersaWrap is placed in the medial and lateral ankle gutters to decrease risk of scar tissue and ectopic bone formation (Fig. 2).

## Results

**Figure 3:**
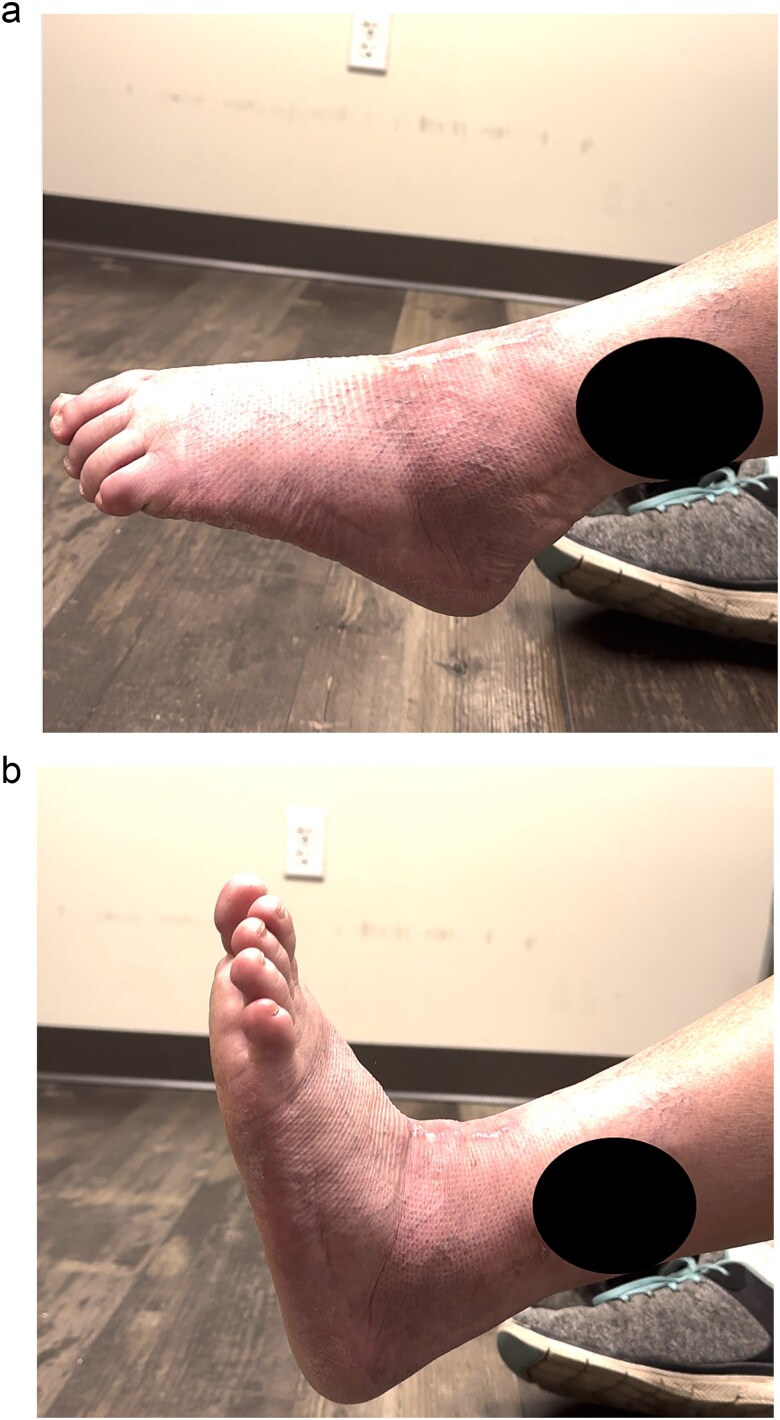
(a and b) Excellent range of motion is maintained post-operatively.

**Figure 4:**
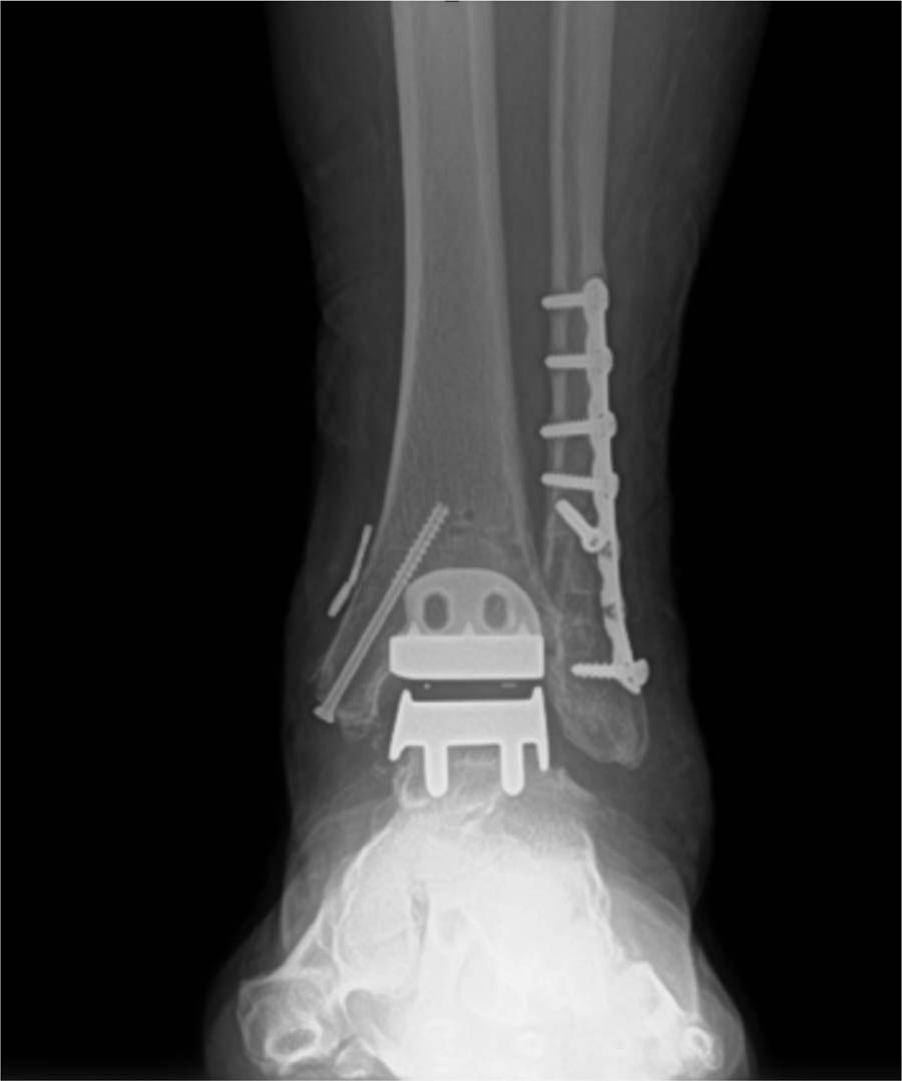
Post-operative radiograph without any sign of gutter impingement or ectopic bone formation.

Excellent maintenance of intra-operative range of motion was observed after surgery (Fig. 3a and b), with significant decreases in pain and no ectopic bone formation noted on radiographs at the most recent follow-up at 1 year post-surgery (Fig. 4). Visual analog pain scale (VAS), American Orthopedic Foot and Ankle Society score (AOFAS), and foot function index (FFI) were recorded before surgery and again at the 1-year follow-up. The pre-operative VAS, AOFAS, and FFI were recorded as 10, 24, and 85.9, respectively. The example patient improved in all measures when compared to pre-operative values, with post-operative VAS, AOFAS, and FFI of 1, 90, and 9.7, respectively.

## Discussion

As total ankle replacements performed throughout the world continue to rise, greater incidences of complications such as postoperative scarring and heterotopic bone will also become more prevalent. Previous methods of treating post-operative total joint scarring have included steroid injection, manipulation either conscious or under sedation, return to surgery for arthroscopic debridement, revision arthroplasty, among others [9, 10]. More recently utilization of local injection of HA has become a treatment option for such adhesions, with good results [11].

This hydrogel HA alginate sheet has been described in literature with successful outcomes for similar purposes in multiple other procedures, such as tendon repair, nerve release, and arthroscopic debridement of the ankle joint [12–14].

Caution should still be utilized with the use of this adjunct, as long-term results are still unknown, and there is limited evidence currently on its use. However, the potential benefits of decreased peri-prosthetic scarring and maintenance of range of motion after total ankle arthroplasty at index surgery could prevent future return to surgery for such issues. The application of this adjunct does not add a significant amount of surgical time and can be applied within seconds.

Utilization of a hydrogel such as VersaWrap at the time of index surgery can potentially decrease the risk of post-operative scarring and heterotopic bone in total ankle replacement, leading to greater range of motion and higher patient satisfaction [6].

## References

[1] McKenna BJ, Cook J, Cook EA, et al. Total ankle arthroplasty survivorship: a meta-analysis. J Foot Ankle Surg 2020;59:1040–8. 10.1053/j.jfas.2019.10.011 32600863

[2] Overley BD Jr, Beideman TC. Painful osteophytes, ectopic bone, and pain in the malleolar gutters following total ankle replacement: management and strategies. Clin Podiatr Med Surg 2015;32:509–16. 10.1016/j.cpm.2015.06.013 26407737

[3] Manegold S, Springer A, Landvoigt K, et al. Heterotopic ossification after total ankle replacement: the role of prosthesis alignment. Foot Ankle Surg 2017;23:122–7. 10.1016/j.fas.2017.02.008 28578795

[4] Schuberth JM, Babu NS, Richey JM, et al. Gutter impingement after total ankle arthroplasty. Foot Ankle Int 2013;34:329–37. 10.1177/1071100712466850 23520289

[5] Chen AF, Lee YS, Seidl AJ, et al. Arthrofibrosis and large joint scarring. Connect Tissue Res 2019;60:21–8. 10.1080/03008207.2018.1517759 30173570

[6] Dekker TJ, Hamid KS, Federer AE, et al. The value of motion: patient-reported outcome measures are correlated with range of motion in total ankle replacement. Foot Ankle Spec 2018;11:451–6. 10.1177/1938640017750258 29277111

[7] St Mart JP, Goh EL, Hay D, et al. Contemporary modern total ankle arthroplasty (TAA): a systematic review and meta-analysis of indications, survivorship and complication rates. Surgeon 2024;22:174–81. 10.1016/j.surge.2024.01.004 38360453

[8] Stoops TK, Sanders RW. Posteromedial accessory incision for posterior capsular release and retractor placement in a total ankle replacement. Foot Ankle Int 2022;43:733–7. 10.1177/10711007211071142 35135339

[9] Zaidi R, Cro S, Gurusamy K, et al. The outcome of total ankle replacement: a systematic review and meta-analysis. Bone Joint J 2013;95-B:1500–7. 10.1302/0301-620X.95B11.31633 24151270

[10] Gross CE, Neumann JA, Godin JA, et al. Technique of arthroscopic treatment of impingement after total ankle arthroplasty. Arthrosc Tech 2016;5:e235–9. 10.1016/j.eats.2015.12.001 27354942 PMC4912567

[11] Marinho A, Nunes C, Reis S. Hyaluronic acid: a key ingredient in the therapy of inflammation. Biomolecules 2021;11:1518. 10.3390/biom11101518 34680150 PMC8533685

[12] Hones KM, Nichols DS, Barker H, et al. Outcomes following use of VersaWrap nerve protector in treatment of patients with recurrent compressive neuropathies. Front Surg 2023;10:1123375. 10.3389/fsurg.2023.1123375 37025263 PMC10071003

[13] Adu Y, Harder J, Cox C, et al. Evaluating the effect of VersaWrap tendon protector on functional outcomes in operative tendon repairs. Front Surg 2024;11:1447515. 10.3389/fsurg.2024.1447515 39737387 PMC11683011

[14] Bridges TN, McCahon JA, Parekh SG. Surgical arthroscopy with intra-articular hyaluronic acid/alginate adjunct in the treatment of ankle osteoarthritis. Techniques in Foot & Ankle Surgery 2024;23:204–7. 10.1097/BTF.0000000000000422

